# Qualitative measurement of opioid effects on pain and dyspnea: gender difference in the sensitivity

**DOI:** 10.1186/s40981-020-00391-y

**Published:** 2020-10-20

**Authors:** Natsuko Nozaki-Taguchi, Taiichiro Hayashida, Shiroh Isono

**Affiliations:** Department of Anesthesiology, Chiba University Graduate School of Medicine, 1-8-1 Inohana Chuo, Chiba, 260-8670 Japan

**Keywords:** Opioid, Analgesia, Dyspnea relieving effect, Experimental pain, Non-respiratory sensation period

## Abstract

**Background:**

An increasing number of patients come to the operating room in use of opioid analgesics. They have different levels of tolerance to opioid effects which challenge the anesthesiologists in search of safe and effective opioid dosing perioperatively. The tested hypothesis is that simple measures introduced will allow us to measure tolerance qualitatively. Opioid effects on pain (analgesia) and dyspnea sensations (relieving effect) are tested. Patients were allocated to three groups according to pre-operative analgesics: (1) control, without any opioid analgesics, (2) weak opioid, and (3) strong opioid. Pressure pain threshold (PPT) and no-respiratory sensation period (NRSP) were measured at two points: before and 3 min after intravenous fentanyl administration.

**Results:**

A total of 58 (43 controls, 9 weak opioids, and 6 strong opioids) patients were enrolled. PPT and NRSP, after iv 2 μg/kg ideal body weight (IBW) fentanyl, were significantly elevated in the control patients (PPT: 6.2 ± 2.1 N to 9.2 ± 3.9 N, *p* < 0.0001, NRSP: 17.8 ± 10.8 s to 22.8 ± 18.7 s, *p* < 0.005, paired *t* test). However, preoperative opioid use, though with tendency, did not show a significant decrease of the opioid effect. Due to an insufficient number of participants, no conclusion could be drawn. Further analysis of the data from control patients showed a significant difference between the two sexes in sensitivity to PPT and NRSP, as well as fentanyl effect on PPT.

**Conclusions:**

Current data showed a simple method of measuring the opioid effect on two dimensions: pain and respiration. Though not able to show a qualitative measurement of tolerance formation in opioid-users, data from control patients showed females to be more sensitive to pain and dyspnea but is less sensitive to the opioid effect. Further studies are necessary to show whether these gender differences serve as clinical relevance.

**Trial registration:**

UMIN, UMIN 000011580. Registered 27 August 2013, https://upload.umin.ac.jp/cgi-open-bin/ctr/ctr.cgi?function=brows&action=brows&type=summary&recptno=R000013352&language=J

## Background

Opioid analgesics have widely been used for not only cancer pain, but also for chronic non-malignant pain. The same opioid drugs are now extensively used during and post-operatively. Not only the variability of surgical stimulus but also the patient’s individual sensitivity to opioid is considered the two major components for successful opioid analgesia. Opioid-consuming patients have a variety of tolerance levels to opioid analgesic effects, probably depending on the dose and duration of their opioid use. In addition, tolerance develops in different manners depending on the opioid effect, such as analgesia, constipation, nausea, and respiratory depression [1]. Tailored pharmacological treatment for post-operative pain has become a further challenge for all anesthesiologists, as preoperative use of an opioid is recently increasing. Pressure pain threshold (PPT) has been reported to rapidly and reliably detect the effect of fentanyl [2]. Whether this simple method allows the detection of tolerance formation has not been reported. As respiratory depression is the most feared complication in opioid analgesia, tolerance to respiratory depression, if detected, will allow us to provide safer analgesia. We applied the PPT test and the measurement of no-respiratory sensation period (NRSP) preoperatively to simultaneously measure the analgesic effect and respiratory depressant effect of fentanyl and development of tolerance.

The study was approved by our institutional ethics committee(#1603: Graduate School of Medicine, Chiba University, Chiba, Japan)and registered in the University Hospital Medical Information Network Clinical Trial Registry (UMIN000011580). Written informed consent was obtained from all patients prior to study enrollment after the aim and potential risks of the study were fully explained.

## Material and methods

ASA 1 or 2 patients for elective surgery from 2015 to 2019 were selected for the study. Patients were grouped to non-opioid users (control), weak-opioid users (weak opioid: either taking tramadol or other opioids ≤ 30 mg oral morphine equivalents), and strong-opioid users (strong opioid: > 30 mg oral morphine equivalents) according to the analgesics used preoperatively. Exclusion criteria were those with respiratory complications, the presence of any skin trouble on the forehead where the pressure test is to be applied and change in opioid dosing within 2 weeks of surgery. In the operating room, ECG, blood pressure, and oxygen saturation were monitored. All patients received 2 L/min oxygen using a nasal cannula. Capnometer (WEC-7301: NIHON KOHDEN, Japan) was attached to the nasal cannula for measurement of respiratory rate.

### Measurement

First, the pressure pain threshold (PPT: Neutone(N)) was obtained by applying an algometer (TAM-1:TRY-ALL, Japan) on the patient’s forehead. The pressure which produced the first pain sensation was measured. Next, the patient was told to stop breathing at end-expiration, and the time-lapse to the first sensation of dyspnea was recorded as no respiratory sensation period (NRSP: second(s)). Both procedures were repeated twice, after which the data were averaged. For safety reasons, PT measurement was terminated at 25 N, and NRSP at SpO_2_ < 90%.

Two parameters were measured pre- and 3-min post intravenous injection of fentanyl 2 μg/kg ideal body weight (IBW). Both PPT and NRSP measurements were explained and practiced once at the ward, after informed consent was taken.

### Statistical analysis

Since we had no basis for the sample size in this observational study, clinical decisions were made according to the availability of patients during the study period. All values are expressed as mean ± SD. All *p* values were two-sided, and a value of *p* < 0.05 was considered statistically significant. Variable changes between pre- and post-fentanyl were compared using Student’s paired *t* test. Analysis of covariance (ANCOVA) was used to analyze the effect of variables on the rate of changes between the groups (primary endpoint). Secondary analysis of control patients was performed, and backward regression analysis was performed to evaluate the predicting factors for PPT and NRSP and also their difference after fentanyl. Linear regression between NRSP and RR changes was also determined.

## Results

A total of 60 patients participated in the study during the 2015 ~ 2019 study period. The original plan was to recruit 90 patients, including 50 controls and 40 opioid users; however, due to the difficulty in recruiting patients consuming a steady amount of analgesics with fair physical status (ASA PS 1 ~ 2) within the study period, the study had to be terminated. Two patients in the control group were eliminated due to the decreased level of consciousness after intravenous fentanyl, resulting in the inability to perform NRSP measurements. Data from 58 patients are included in the final assessment. Demographic data for 43 control, 9 weak opioid, and 6 strong opioid groups are shown in Table 1 with no statistical difference between the groups. Patients with strong opioids were all, excluding 1, cancer patients with progressive disease, while those with weak opioids were non-cancer chronic disease patients.

**Table 1:** Patient demographic

	Control	Weak opioid		Strong opioid	
n	43	9		6	
Age	57.1 ± 16.4	54.6 ± 16.8		59 ± 12.3	
Sex	M18 F25	M3 F6		M3 F3	
BMI (kg/m 2)	22.6 ± 3.3	23.7 ± 4.3		20.7 ± 3.0	
PS	PS1:20 PS2:23	PS1:4 PS2:5		PS2:5	
With pain (%)	32.6	100		100	
Opioids	None	tramadol	8	oxycodone > 15 mg	1
		oxycodone < 10 mg	1	fentanyl	4
				methadone	1
Oral morphine equivalents (mg)	0	22.5 (7.5 ~ 45)		323 (75 ~ 858)	
Nsaids	7	4		2	

### PPT, NRSP difference, and pre-operative opioid use (primary endpoint of the study)

**Fig. 1:**
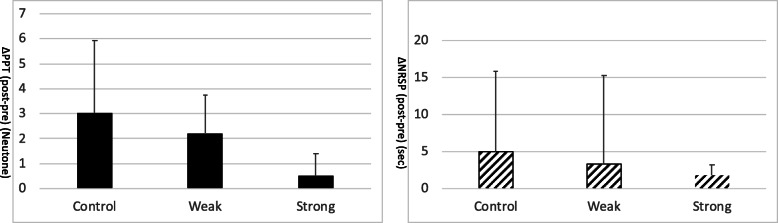
PPT difference (ΔPPT (post-pre)) (left) and NRSP difference (ΔNRSP (post-pre)) (right) amongst the three patient group (mean ± SD) were not significant but showed tendency to decrease with pre-operative opioid (ANCOVA *p* > 0.15 for PPT, *p* > 0.9 for NRSP)

PPT and NRSP change after fentanyl administration was compared among the three groups, as to detect the presence of tolerance to the opioid effect. PPT difference (ΔPPT post-pre) and NRSP difference (ΔNRSP post-pre), though with tendency, no significant correlations were detected with pre-operative opioid use (Fig. 1: ANCOVA *p* > 0.15 for PPT, *p* > 0.9 for NRSP). Unfortunately, with the limited participants in opioid users, we could not detect any relevant effect changes. However, simultaneous measurement of analgesic and respiratory depressant effect with experimental measures showed interesting results and further analysis was performed only for control patients.

### PPT and NRSP measurement in control patients

In the control patients (*n* = 43), PPT increased from 6.2 ± 2.1 N to 9.2 ± 3.9 N after iv fentanyl 2 μg/kg IBW administration (*p* < 0.0001, paired *t* test), showing the analgesic effect of fentanyl. Backward regression analysis was performed by using 7 background variables (age, sex, BMI, physical status (PS), pre-operative mean blood pressure, heart rate, and respiratory rate) to determine individual predictors for pre-operative PPT and PPT difference (ΔPPTpost-pre). The female sex was identified as a statistically significant predictor for pre-operative PPT (female: 5.6 ± 2.1 N vs male 7.0 ± 1.7 N, *p* = 0.028) while female sex, BMI, and PS were identified as statistically significant independent predictors for ΔPPT (post-pre) (sex *p* = 0.03, BMI *p* = 0.007, PS *p* = 0.03).

NRSP in the control group was 17.8 ± 10.8 s which increased to 22.8 ± 18.7 s after fentanyl iv (*p* < 0.005, paired *t* test) without any significant decrease in SpO_2_, showing dyspnea relieving effect, in another word, respiratory depressive effect of fentanyl. The same 7 background variables were used to detect predictors for pre-operative NRSP: sensitivity for dyspnea and ΔNRSP (post-pre): sensitivity for fentanyl on respiratory effect. Female sex was again identified as a statistically significant predictor of NRSP (female: 14.4 ± 9.0 s vs male 22.5 ± 11.7 s, *p* = 0.01). However, the fentanyl depressive effect on NRSP difference (ΔNRSP (post-pre)) was not correlated with any of the variables. Six background variables were compared between male (*n* = 18) and female (*n* = 25) and found no statistical difference (Table 2).

**Table 2:** Patient demographic by sex

	Male		Female	
n	18		25	
Age (yr)	61.7 ± 14.7		53.8 ± 17.1	
PS	1:6	2:12	1:14	2:11
BMI (kg/m 2)	23.3 ± 3.4		22.2 ± 3.2	
mean BP (mmHg)	100.2 ± 10.0		99.5 ± 13.2	
HR (/min)	68.2 ± 12.0		73.81 ± 11.9	
RR (/min)	18.5 ± 4.0		17.1 ± 3.8	

### Vital signs 3 min after fentanyl administration

Three minutes after fentanyl administration, HR and RR significantly decreased in the control group (71 ± 12 to 68 ± 12 bpm, 18 ± 3.9 to 13 ± 3.8/min, respectively: paired *t* test *p* < 0.0001). No clinically relevant decrease in the vital sign was observed in all patients including oxygen saturation with 2 L supplemental oxygen. None of the three vital sign changes showed a gender difference. When comparing two respiratory measures, a decrease in RR had no significant correlation with ΔNRSP (*p* > 0.9, *R* = 0.019).

## Discussion

### Experimental measurement of opioid tolerance

In this study, our first plan was to measure opioid (fentanyl) effect on two modalities, analgesia and dyspnea relieving effect, and apply these methods to detect opioid tolerance qualitatively, so as to provide an effective and safe analgesia peri-operatively in opioid-consuming patients. Not all patients with opioid analgesia develop tolerance at the same level. The duration of the dosing is likely to play an important role; however, individual sensitivity to opioid also differs amongst patients. Accordingly, this simple method may give us a reliable guide for us anesthesiologists for correct dosing.

Several studies have tried to qualitatively measure the opioid effect with experimental pain [2, 3]; however, simultaneous measurements of its effect on respiration have not been reported. We were able to evaluate the fentanyl analgesic effect with an elevation of PPT and effect on respiration with elongation of NRSP. Unfortunately, with a limited number of participants, no statistical difference could be observed in opioid users, such that measurement of tolerance to the opioid effect needs to be re-studied.

### Simultaneous measurement of opioid effect on pain and respiration

PPT measurement was quick, reliable, and non-invasive. NRSP measurement was also non-invasive and easy to measure without any equipment. The total time to perform the two measurements was within 5 min, and with supplemental 2 L oxygen, no case was observed with SpO_2_ decrease below 90%. NRSP is known to show a significant correlation to CO_2_ ventilatory chemosensitivity [4, 5]. As morphine has been shown to depress hypercapnic ventilatory response [6], we expected NRSP to be a suitable measurement for the opioid effect on respiration. We actually did observe an increase in NRSP after fentanyl administration showing a relieving effect of fentanyl on dyspnea sensation which has not been reported anywhere. Another easily measured respiratory modality is RR which also showed a significant decrease after iv fentanyl; however, this decrease did not correlate with NRSP change. Whether this difference between the two modalities indicates a different mechanism of respiratory depression, further study is necessary. However, this fact alerts us that RR measurement may not be enough to detect respiratory depression induced by fentanyl.

### Gender difference in perception and opioid effect

Our data demonstrates that there is a significant sex difference in the perception of pain and also dyspnea. Female tendency for being sensitive to pain has been reported with several experimental measures [7]. However, females being less effective to opioid analgesics have competing results, especially when measured in clinical settings. More side-effects observed in females tend to decrease opioid consumption [8]. Our data also showed female to have decreased threshold to NRSP, showing sensitivity to dyspnea sensation. Though we were not able to detect a difference between the two genders in NRSP changes after fentanyl at our dose, differences between men and women in the way morphine affects the ventilatory responses to carbon dioxide and oxygen have been reported in a small number, healthy volunteer study [9]. Since most studies on the respiratory effect of opioids have been performed with morphine, there may be a difference amongst the variable opioids used, since fentanyl effect on relieving dyspnea is reported to be less clear compared with morphine clinically [10].

## Limitations

NRSP measurement was easy to perform without any equipment and with the least risk to the patient. However, unlike pain sensation, to some patients, the meaning of the first sensation of dyspnea seemed difficult to comprehend and tended to perform breath-holding close to maximum. These may explain the wide range of individual variability observed in NRSP measurement. However, in most cases, in those with longer NRSP, repeated measurement showed similar results such that the change rate after fentanyl is likely to be reliable.

As we could not recruit enough patients under opioid analgesia in a constant dosing with fair physical status, no statistically significant results could be drawn. Patient selection criteria may need to be changed. However, during this study period, many patients under opioid analgesia came to the operating room for surgical treatment. Among these patients, there were those whose opioids were either recently started, increased pre-operatively, changed from oral to intravenous intake, or switched types due to pain escalation before surgery. These unfortunately did not meet our inclusion criteria, however needed special care for perioperative opioid dosing. If we were able to measure the level of tolerance to these patients, more rationale use of intra and postoperative dosing for opioid may be planned. Accordingly, we believe that these measures are clinically important and need further investigation.

## Conclusion

Preoperative simple measurement for fentanyl effect on pain and respiration was proposed. If sensitivity to two modalities may be measured preoperatively, safer and efficacious peri-operative analgesic protocol may be possible, not only in opioid-tolerant patients but also in all patients to be treated. The sex difference in the effect of the opioid effect needs further studies to elucidate its importance in the clinical practice.

## Data Availability

The datasets used and/or analyzed during the current study are available from the corresponding author on reasonable request.

## Abbreviations

PPT: Pressure pain threshold
NRSP: No-respiratory sensation period
